# Neighborhood disadvantage is associated with *KRAS*-mutated non-small cell lung cancer risk

**DOI:** 10.1007/s00432-022-04455-7

**Published:** 2022-11-16

**Authors:** Sam E. Wing, Marta M. Jankowska, Xiaoke Zou, Ernesto Sosa, Jiue-An Yang, Tarik Benmarhnia, Susan L. Neuhausen, Rebecca Nelson, Ravi Salgia, Stacy W. Gray, Loretta Erhunmwunsee

**Affiliations:** 1 Department of Population Sciences, City of Hope Comprehensive Cancer Center, 1500 E Duarte Rd, Duarte, CA 91010, USA; 2 Herbert Wertheim School of Public Health, University of California San Diego, San Diego, CA, USA; 3 Scripps Institution of Oceanography - Climate, Atmospheric Sciences, and Physical Oceanography, San Diego, CA, USA; 4 Department of Computational and Quantitative Medicine, City of Hope Comprehensive Cancer Center, Duarte, CA, USA; 5 Department of Medical Oncology & Therapeutics Research, City of Hope Comprehensive Cancer Center, Duarte, CA, USA; 6 Department of Surgery, City of Hope Comprehensive Cancer Center, Duarte, CA, USA

**Keywords:** *KRAS* mutations, Health disparities, Neighborhood socioeconomic status, Non-small cell lung cancer, Molecular epidemiology

## Abstract

**Purpose:**

It remains unclear why individuals living in disadvantaged neighborhoods have shorter non-small cell lung cancer (NSCLC) survival. It is possible that living in these deprived areas is linked with increased risk of developing aggressive NSCLC biology. Here, we explored the association of somatic *KRAS* mutations, which are associated with shorter survival in NSCLC patients, and 11 definitions of neighborhood disadvantage spanning socioeconomic and structural environmental elements.

**Methods:**

We analyzed data from 429 NSCLC patients treated at a Comprehensive Cancer Center from 2015 to 2018. Data were abstracted from medical records and each patient’s home address was used to assign publicly available indices of neighborhood disadvantage. Prevalence Ratios (PRs) for the presence of somatic *KRAS* mutations were estimated using modified Poisson regression models adjusted for age, sex, smoking status, race/ethnicity, educational attainment, cancer stage, and histology.

**Results:**

In the NSCLC cohort, 29% had *KRAS* mutation-positive tumors. We found that five deprivation indices of socioeconomic disadvantage were associated with *KRAS* mutation. A one decile increase in several of these socioeconomic disadvantage indices was associated with a 1.06 to 1.14 increased risk of *KRAS* mutation. Measures of built structural environment were not associated with *KRAS* mutation status.

**Conclusion:**

Socioeconomic disadvantage at the neighborhood level is associated with higher risk of *KRAS* mutation while disadvantage related to built environmental structural measures was inversely associated. Our results indicate not only that neighborhood disadvantage may contribute to aggressive NSCLC biology, but the pathways linking biology to disadvantage are likely operating through socioeconomic-related stress.

## Introduction

Approximately 135,720 Americans died from lung cancer in 2020 (National Cancer Institute 2020), making it the leading cause of cancer death in the United States (US). A disproportionately high number of cases and deaths occur in individuals of low socioeconomic status (SES), due to various mechanisms, including late-stage diagnoses, less access to treatment, decreased survival (Forrest et al. 2013; Finke et al. 2018), and greater tumor progression (Peterson et al. 2014; Percy-Laurry et al. 2018; Guerrero-Preston et al. 2019). Differences in cigarette smoking patterns may be responsible for a portion of these disparities (Pinsky 2006), but inequities persist after controlling for smoking and access to care (Forrest et al. 2013; Johnson et al. 2014). Thus, to adequately address lung cancer burden in the US, it is critical to identify additional sources of disparity.

Because low SES individuals often live in neighborhoods with adverse conditions (Roux et al. 2001). it is important to consider the impact of the neighborhood environment on lung cancer. In a sample of 3.2 million Swedish adults, individuals living in the most deprived neighborhoods had 27% and 32% higher odds of developing and dying of lung cancer, respectively (Li et al. 2015). Other studies have found that ecological factors play an important role in cancer incidence and exacerbate existing inequities in cancer outcomes (Erhunmwunsee et al. 2012; Ellis et al. 2018; Adie et al. 2020).

Exposure to adverse neighborhood conditions may increase the risk of developing aggressive NSCLC biology (Erhunmwunsee et al. 2021). Fifteen to 40% of NSCLCs have somatic mutations in *KRAS* (vi-Ki-ras2 Kirsten rat sarcoma viral oncogene). *KRAS* is in a family of guanosine triphosphate binding proteins that play essential roles in regulating normal cellular proliferation and cell signaling (Vojtek and Der 1998). *KRAS* somatic mutations are biomarkers of aggressive tumor biology (Graziano et al. 1999; Wood et al. 2016) and are associated with worse survival rates and faster recurrence among NSCLC patients (Mascaux et al. 2005; Gautschi et al. 2007). Although *KRAS*-mutated tumors are resistant to some targeted therapies, they are responsive to immunotherapy with a targeted therapy available for *KRAS(G12C)* (Amanam et al. 2020; Skoulidis et al. 2021). Despite the increased focus on treatment opportunities for *KRAS*-mutated NSCLC, there remains a lack of understanding of how adverse neighborhood conditions may promote its development.

Disadvantage can be measured in several ways. The same neighborhood might be classified as disadvantaged according to one metric but less disadvantaged according to another (Ortiz et al. 2020). Furthermore, commonly used neighborhood deprivation measures like the Area Deprivation Index (ADI) (Kind and Buckingham 2018) focus on measures of composition, such as aggregate metrics of individual poverty, rather than evaluating neighborhood context, such as areas that are safe for walking and access to jobs (Macintyre et al. 2002). These are important distinctions. Some previous research have suggested that a neighborhood’s environmental, physical, and social contexts have the greatest influence on health (contextual measures) (Macintyre et al. 1993), whereas others have posited that an area’s health is more reflective of the composition of the population living there (compositional measures) (Sloggett and Joshi 1994). It has been estimated that contextual measures are responsible for about 60% of a neighborhood’s impact on physical impairment with the remainder being attributable to compositional factors (Ross and Mirowsky 2008). Thus, understanding how metrics of area-level disadvantage are related to NSCLC biology is a vital precursor to interventions that address disproportionately high rates of NSCLC and poor outcomes among communities of low SES.

This study addresses these limitations by utilizing 11 available indices incorporating various social determinants of health, including education access, social and community context, economic stability, and the built environment. We aimed to identify whether measures of neighborhood disadvantage are associated with *KRAS* mutations to gain insight into area-level factors that place individuals at higher risk of aggressive NSCLC. This study provides a novel assessment of the influence of neighborhood disadvantage on the presence of somatic *KRAS* mutations in NSCLC patients.

## Materials and methods

### Study participants

We included all patients with NSCLC who were treated at a comprehensive cancer center from 2015 to 2018. Inclusion criteria were: (1) a primary diagnosis of NSCLC and (2) *KRAS* somatic sequencing as documented in the electronic health record (EHR). Exclusion criteria were: (1) diagnosis of small cell lung cancer, carcinoid, or sarcomas; (2) in situ lung cancer; (3) age < 18 years; (4) missing covariates; and/or (5) multiple primary NSCLCs with different somatic phenotypes. The Institutional Review Board approved all study activities and issued a waiver of documentation of informed consent (#18257).

### Patient-level measures

We obtained age at diagnosis, sex, race/ethnicity, stage and histology, smoking history, education, and address from the institutional cancer registry. Smoking history was collected from clinic notes and/or patient surveys. Somatic testing was performed as part of clinical care and included sequencing of tumor and/or blood-based assays. Approximately 90% of tests were conducted by either the institutional Clinical Molecular Diagnostics Laboratory, Foundation Medicine, Inc. (San Diego, CA), or Guardant Health, Inc. (Redwood City, CA). We obtained *KRAS* mutation status and the corresponding subtypes (G12A, G12A Amp, G12C, G12D, G12F, G12R, G12S, G12V, G13C, G13D, K117N, L19F, Q61H and Q61L) through medical record abstraction. We included test results from multiple samples for each patient. For patients with discrepant results, findings from tissue were prioritized over those from blood-based assays (cell-free DNA).

### Neighborhood-level measures

Neighborhood disadvantage scores were defined using 11 indices from various national and state agencies (Table 1): the Area Deprivation Index (ADI), California Healthy Places Index (HPI), Jobs Proximity Index (Jobs Prox), Labor Market Engagement Index (Labor), Low Poverty Index, Low Transportation Cost Index (LTCI), Regional Opportunity Index (ROI): People, ROI: Place, School Proficiency Index (Sch. Prof.), Social Vulnerability Index (SVI), and Walkability Index. The indices are freely available through online portals and have been previously validated.

The US Department of Housing and Urban Development (HUD) developed and distributed five indices to aid in the tracking and enforcement of the Fair Housing Act (Gourevitch et al. 2018): three of the HUD indices focus on contextual neighborhood characteristics (job access [Jobs Prox.], transportation opportunities [LTCI], and school proficiency [Sch. Prof.), while two focus on compositional factors (poverty [Poverty Index] and labor market engagement [Labor]). The indices have been used to evaluate racial/ethnic disparities in 49 local government jurisdictions (Housing and Urban Development Department 2018).

The ADI, developed by the University of Wisconsin School of Public Health, ranks neighborhood deprivation based on income, education, employment, and housing quality (Kind et al. 2014). The ADI has been used to study poverty-based deprivation and its association with lung cancer (Fairfield et al. 2020). The HPI, developed by the Public Health Alliance of Southern California, is a weighted composite of factors associated with life expectancy in the state (Maizlish et al. 2019) and has been used to close health gaps among underserved communities (Public Health Alliance of Southern California 2020). The ROI, developed at the University of California Davis Center for Regional Change, measures a neighborhood’s educational, economic, housing, transportation, health, and civic assets. This index is divided into a “people” measure focusing on the individuals within an area, taking a compositional framework; and a “place” measure focusing on the institutions within the area, taking a contextual framework. The Center for Disease Control’s SVI estimates a neighborhood’s need for support relative to a hypothetical hazardous disaster event and relies mostly on compositional census data, with one component focused on the types/sizes of housing structure types in each census tract (Agency for Toxic Substances and Disease Registry 2020). Finally, the Walkability Index from the Environmental Protection Agency ranks a neighborhood’s built environment based on the likelihood that it promotes walking as a main mode of transportation (U.S. Environmental Protection Agency 2020). The index is created from contextual neighborhood characteristics. A breakdown of each index and components included is in Supplemental Table 1.

Indices were computed for the institutional catchment area, including Los Angeles, Orange, Riverside, and San Bernardino counties in California. All indices were aggregated at the census tract level and transformed or re-ordered as needed to a 0 to 10 scale, where 0 was the best outcome/least disadvantage and 10 was the worst outcome/most disadvantage. Each patient’s home address was geocoded, and the associated census tract was used to assign the 11 index values.

### Statistical analysis

The primary variables of interest were the 11 neighborhood disadvantage indices. Correlations between index values for all census tracts in the study area were calculated using a Pearson correlation. Descriptive statistics for the sample were computed, and differences by *KRAS* mutation status were examined using t-test for continuous variables and chi-squared tests for nominal/categorical data. To assess the association between neighborhood disadvantage and *KRAS* mutation status, we fit a Poisson regression model with robust error variance (because of high prevalence for the outcome) to estimate Prevalence Ratios (PR) and their 95% confidence intervals (CIs) for each index (Zou 2004; Knol et al. 2012). Models were adjusted for covariates based on a priori knowledge of their status as possible confounders, including sex, age at diagnosis, race/ethnicity, educational attainment, cancer stage and histology, and smoking history. All analyses were performed using SAS 9.4 (SAS Institute, Cary, NC).

## Results

**Fig. 1:**
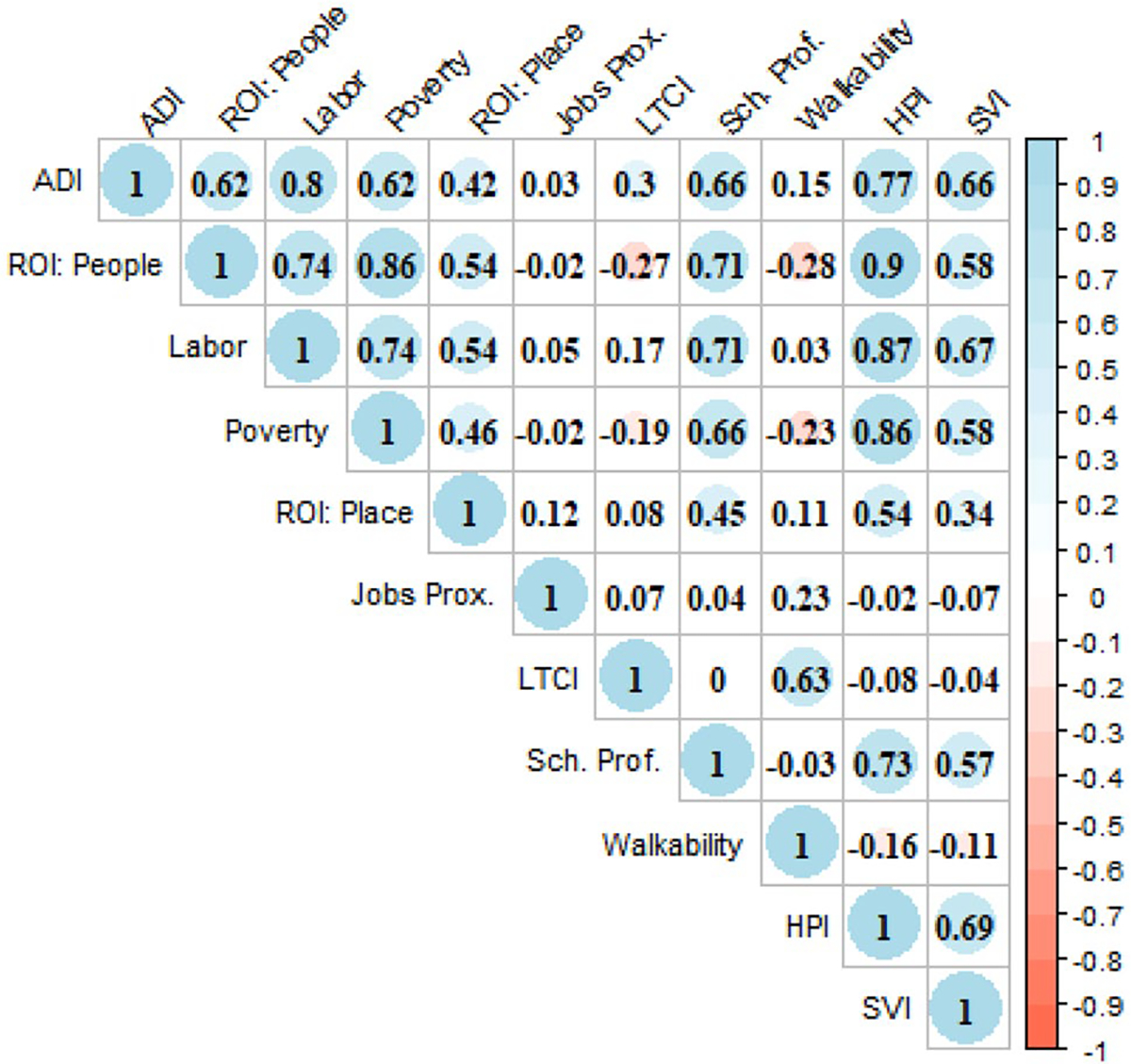
Correlations between the 11 neighborhood disadvantage indices. Pearson’s correlation matrix of neighborhood disadvantage indices measured in census tracts of the study area. Size of the circle indicates absolute value of the correlation, while color indicates negativity and positivity of the correlation

Associations between the 11 neighborhood disadvantage indices were mixed (Fig. 1). There were high correlations among compositional measures, up to *r* = 0.85 between Poverty and ROI: People indices. Correlations were much lower between contextual measures. The highest correlation was between Walkability and Transportation cost (*r* = 0.63), followed by Sch. Prof. and ROI: Place (*r* = 0.45). Other correlations were below *r* = 0.21. The highest correlation between compositional and contextual measures was between Sch. Prof. and all compositional measures (all > *r* = 0.64). HPI and SVI, which include compositional and contextual measures, correlate highly across measures except for Jobs Proximity, LTCI, and Walkability.

Of 541 eligible NSCLC patients with somatic *KRAS* testing, 429 were included in the analysis. Due to missing baseline address or essential covariate data, including race, histology, smoking status, or education level, 112 were excluded. Patients were predominantly female (51%), former or current smokers (62%), non-Hispanic White (56%), and had obtained less than a four-year college degree (58%). Most patients had stage III or IV disease (86%) and adenocarcinoma (86%). *KRAS* mutations were detected in 29% of patients (*n* = 124), with prevalence varying significantly across race/ethnicity (higher in non-Hispanic White and lower in Asian patients), stage (higher in stages I-III and lower in stage IV), histology (higher in adenocarcinomas and lower in squamous cell tumors), and smoking status (higher in former and current smokers) (Table 2).

In crude models, 2 of the 11 neighborhood deprivation indices were positively and precisely associated with the presence of a *KRAS* mutation (Table 3). For both Labor and Sch. Prof., a one decile increase (i.e., more disadvantage) was positively associated with *KRAS* mutation (PR = 1.07 95% CI: 1.01, 1.13). In fully adjusted models, we found a similar pattern. Furthermore, we found that greater disadvantage as measured through ADI, ROI: People, and the HPI indices were associated with increased risks of *KRAS* mutation (PR = 1.06, 95% CI: 1.01, 1.12; PR = 1.14, 95% CI: 1.01, 1.30; and PR = 1.08, 95% CI: 1.01, 1.15 respectively). Conversely, Jobs. Prox. was negatively associated with risks of *KRAS*. For each decile increase (i.e., less proximity to jobs), there was a 0.93 lower risk of *KRAS* mutation (95% CI: 0.87, 1.00). This inverse association was also seen for the ROI: places and walkability, albeit with lower precision.

## Discussion

This study found a consistent increased risk of *KRAS* mutation, up to 1.14 times more risk, for individuals living in more socioeconomically deprived census tracts. In this exploratory analysis, we found evidence that socioeconomic disadvantage at the neighborhood-level is associated with higher risk of *KRAS* mutation, while environmental structural disadvantage was not associated or negatively associated. These findings indicate that neighborhood disadvantage may contribute to aggressive NSCLC biology and that the pathways linking biology to neighborhood disadvantage are likely operating through compositional and contextual socioeconomic-related stress. Our findings support using a wide range of disadvantage variables that provide both compositional and contextual perspectives in evaluating neighborhoods on lung cancer outcomes.

Several measures of neighborhood disadvantage were positively linked to risk of *KRAS* mutation, including the ROI: People, Labor, ADI, HPI and Sch. Prof. Most of these measures are compositional in nature (except for Sch. Prof.) and all provide some assessment of income, employment and/or educational attainment—which have all been shown to significantly impact individual and population health (Sloggett and Joshi 1994). Each of these indexes emphasizes slightly different socioeconomic aspects (see supplemental table). For example, the ROI: People, ADI, and HPI include income, employment, educational attainment, home ownership, the cost/value of housing, and transportation access categories. The Labor index also had a positive association with the risk of *KRAS* mutation; however, this index is not as comprehensive as the ROI: People, ADI or HPI. Instead, the Labor index combines only three socioeconomic factors: unemployment rate, bachelor’s degree attainment and labor-force participation rate. Unemployment rate and educational attainment have been directly linked to NSCLC mortality (Erhunmwunsee et al. 2012; Antunes 2016; Hagedoorn et al. 2016).

Sch. Prof. was the sole contextual index positively associated with *KRAS* mutation status. This index measures school performance and resources—another estimate of education. The consistent relationship between the presence of *KRAS* mutation with five disadvantage indices—all of which included educational attainment/access estimation and four of which included employment assessment—indicates the importance of specific domains of neighborhood composition on health outcomes, as well as the importance of relative measures that assess inequity of a neighborhood compared to surrounding areas. Lack of appropriate educational and employment opportunities may be associated with psychosocial stress that leads to poor health outcomes (van der Noordt et al. 2014) and upstream immune changes related to the acquisition of somatic mutations (Reiche et al. 2005). Furthermore, socioeconomic neighborhood disadvantage is associated with poverty, social stress, unsafe neighborhoods, and lack of healthcare access that can promote comorbid diseases, which may adversely contribute to the aggression of cancer biology (Prakash et al. 2020).

To date, there has been no investigation exploring how or whether neighborhood socioeconomic factors impact lung cancer biology as an explanation of how adverse neighborhood conditions predict NSCLC mortality. We previously found that elevated PM_2.5_ levels were linked to increased risk of *TP53* somatic mutations (Erhunmwunsee et al. 2021), which, like *KRAS* somatic mutations, are associated with worse NSCLC mortality. The findings from the current study continue to support the idea that neighborhood socio-environmental context may impact the risk of aggressive biology, thus contributing to worse outcomes.

We found an inverse relationship between neighborhood-built environment/infrastructure and aggressive lung cancer biology. For example, the Jobs Prox. was negatively associated with *KRAS* mutations, i.e., living further from job opportunities was linked to lower *KRAS* mutation. Although there may be many jobs in areas of the inner city where individuals with low SES reside, proximity to jobs may not reflect access to career opportunities that promote economic advancement. Our findings that socioeconomic disadvantage is associated with *KRAS* mutation, while built environment disadvantage was not as strongly, suggests that the stress of low income/education/employment may outweigh the stress of lack of infrastructure amenities, namely because the people who lack such amenities (those who do not live in the inner city) typically have enough resources to not need them.

It remains unclear whether individual or neighborhood SES may contribute to the development of *KRAS* NSCLC directly or through other mediating biological processes like inflammation (Kitajima et al. 2016). While this study provides evidence linking disadvantaged areas with *KRAS* mutation status, additional research into the relationships between individual and community-level disadvantage and the biological mechanisms that lead to aggressive NSCLC is needed. There is evidence supporting associations of low individual SES and elevated levels of inflammatory markers (Koster et al. 2006) and inflammatory/immune status (Castagné et al. 2016). Furthermore, low individual SES and neighborhood-related social determinants of health have been linked to epigenetic aging, measured by DNA methylation (Guerrero-Preston et al. 2019). DNA methylation, in turn, has been associated with the presence of *KRAS* mutations in colorectal and lung cancer (Nagasaka et al. 2004; Bjaanæs et al. 2016). Despite these links, the pathways through which individual and neighborhood-level SES factors contribute to aggressive tumor biology have not been established. In this study, we elucidate an association between several measures of neighborhood socioeconomic disadvantage and somatic *KRAS* mutations in lung cancer patients. However, more research and larger numbers are needed to establish causation and the pathways involved, which will ultimately support treatment and intervention efforts to eliminate disparities in NSCLC.

Our results did not support the assumption that the negative impact of neighborhood disadvantage on lung cancer biology is solely attributable to increased smoking among marginalized groups. Even after controlling for tobacco use, NSCLC patients who lived in neighborhoods with lower Labor, ROI: People, Sch. Prof., ADI, and HPI scores had higher odds of a *KRAS* mutation. Although we cannot completely rule out residual confounding by smoking, this result suggests that the impact of the neighborhood environment on mutational changes extends beyond tobacco exposure. These findings provide further evidence that neighborhood conditions significantly impact health, independent of smoking. Efforts to improve neighborhoods at the economic, social, education, housing and transportation levels are paramount in addition to our continued efforts to lower smoking exposure.

Importantly, rather than using only one disadvantage index, we selected 11 indices previously used in national and state settings to evaluate the influence of compositional and contextual elements of neighborhood-level disadvantage. Recent research on neighborhood disadvantage and cancer outcomes highlighted the need to utilize multiple measures to identify the factors contributing to cancer burden and inform future prevention efforts (Ortiz et al. 2020). Pairwise correlations between the neighborhood measures showed large variation in the level of correlation across measures. It is important to note that we do not adjust for multiple testing due to the exploratory nature of the study, and because the dataset was not collected with our stated hypotheses in mind (Bender and Lange 2001). The results found here are exploratory and require future confirmatory studies designed to assess the impact of neighborhood disadvantage on lung cancer somatic mutations and include methods that would consider the synergistic effects of such neighborhood measures.

Our study had several strengths, including being the first to directly examine the relationship between neighborhood disadvantage and *KRAS* mutations in NSCLC. This investigation was possible due to access to clinical somatic data, not typically available in larger cancer registries. The EHR data enabled control for individual-level SES factors and risk factors like smoking. The study had some weaknesses. Due to the clinical nature of the data, the sample size was small and there was limited racial/ethnic variation in the sample. Future research may seek to recruit patients from underrepresented populations to better understand the role of race, ethnicity, and disadvantage in NSCLC biology and outcomes. This is especially important as racial/ethnic minorities tend to reside in neighborhoods with worse conditions than their non-Hispanic White counterparts (Osypuk et al. 2009). Additionally, each patient’s home address was obtained at the time of their diagnosis without information about how long they had lived there. There is a significant gap in the literature about timing related to the development of somatic mutations in NSCLC. Future studies should consider residential histories to better understand how neighborhood disadvantage and environment play a role in the timeline of *KRAS* mutation development. Finally, we lacked information on the details of other mutations in this cohort. Further analysis of concomitant mutations and immune biomarkers would broaden our understanding of the impact of socioenvironmental context on tumor biology. Future studies should include information about the ancestral variation of *KRAS* mutations and codon-level detail about these and other mutations. There remains a need to determine how *KRAS* subset differences correlate with smoking status, pollution, neighborhood disadvantage, race, SES, ancestry, and other genomic alterations. Lastly, future studies should assess how race, disadvantage, and somatic *KRAS* mutations contribute to survival differences.

Although neighborhood disadvantage has been associated with the aggressive biology of some cancers, there is less evidence that the same relationship exists for NSCLC. This study expands the literature by demonstrating that, in our sample of NSCLC patients, several measures of socioeconomic neighborhood disadvantage were significantly associated with *KRAS* mutations. Understanding the mechanisms that link neighborhood disadvantage to aggressive NSCLC biology in vulnerable populations is vital to develop strategies to overcome disparities in NSCLC.

## Supplementary Material

Supplementary Table 1

## Figures and Tables

**Table 1:** Neighborhood deprivation measures, descriptions, and sources. Further description of each index and factors included are in Supplemental Table 1

Index	Description	Compositional or Contextual	Source

Area deprivation	Ranking of neighborhood socioeconomic disadvantage, compared within CA	Compositional	Univ. of WI Neighborhood Atlas
Regional opportunity index: people	Measure of people’s assets in education, economy, housing, transportation, health, and civic life	Compositional	UC Davis Center for Regional Change
Labor market engagement	The relative intensity of labor market engagement and human capital in a census tract: level of employment, labor force participation, and educational attainment	Compositional	U.S. Department of Housing and Urban Development (HUD)
Poverty	The poverty rate within a given census tract	Compositional	HUD
Regional opportunity index: place	Measure of a place’s assets in education, economy, housing, transportation, health, and civic life	Contextual	UC Davis Center for Regional Change
Jobs proximity	Measure of the accessibility of a residential neighborhood to all job locations within a corebased statistical area (CBSA)	Contextual	HUD
Low transportation cost	Measure of the availability of low-cost transportation options within a given census tract	Contextual	HUD
School proficiency	State exam scores of 4th-grade students in local elementary schools	Contextual	HUD
Walkability	Ranking of an area’s built environment characteristics that influence the likelihood of walking being used as a mode of travel	Contextual	Environmental Protection Agency
California healthy places	Weighted composite of factors predicting life expectancy in CA, including economic, social, education, transportation, neighborhood, housing, clean environment, and health care	Both	Public Health Alliance of Southern CA
Social vulnerability	Measure to identify communities that will most likely need support before, during, and after a hazardous event based on social factors, including poverty, lack of vehicle access, and crowded housing	Both	Centers for Disease Control

**Table 2:** Demographic characteristics for the cohort and by *KRAS* mutation status

Characteristic	Overall, *N*=429^1^	KRAS Mutation Negative, *n* = 305^1^	KRAS Mutation Positive, *n* = 124^1^	*p*-value^2^

Sex				0.2
Female	217 (51%)	161 (53%)	56 (45%)	
Male	212 (49%)	144 (47%)	68 (55%)	
Educational attainment				0.2
< HS Grad	46 (11%)	32 (10%)	14 (11%)	
College Degree	117 (27%)	91 (30%)	26 (21%)	
Grad Degree	64 (15%)	47 (15%)	17 (14%)	
HS Grad	202 (47%)	135 (44%)	67 (54%)	
Insurance status				0.8
Medicaid	29 (6.8%)	20 (6.6%)	9 (7.3%)	
Not Med	400 (93%)	285 (93%)	115 (93%)	
Race/Ethnicity				0.015
Asian	133 (31%)	108 (35%)	25 (20%)	
Black	18 (4.2%)	12 (3.9%)	6 (4.8%)	
Hispanic-White	37 (8.6%)	27 (8.9%)	10 (8.1%)	
Non-Hispanic White	241 (56%)	158 (52%)	83 (67%)	
Stage				0.024
1–2B	62 (14%)	38 (12%)	24 (19%)	
3A/3B	67 (16%)	42 (14%)	25 (20%)	
4	300 (70%)	225 (74%)	75 (60%)	
Histology				0.013
Adenocarcinoma	369 (86%)	255 (84%)	114 (92%)	
Other	28 (6.5%)	20 (6.6%)	8 (6.5%)	
Squamous	32 (7.5%)	30 (9.8%)	2 (1.6%)	
Smoking Status				< 0.001
Current smoker	67 (16%)	39 (13%)	28 (23%)	
Former smoker	199 (46%)	127 (42%)	72 (58%)	
Never smoker	163 (38%)	139 (46%)	24 (19%)	
Age	67 (12)	67 (13)	69 (10)	0.073
PM2.5 exposure, ug/m^3^	11.58 (1.59)	11.60 (1.53)	11.51 (1.74)	> 0.9

1 *n* (%); Mean (SD)

2 Pearson’s Chi-squared test; Fisher’s exact test; Wilcoxon rank sum test

**Table 3:** Crude and adjusted prevalence ratios (PR) with 95% Confidence Intervals (CI) of having *KRAS* mutation based on neighborhood disadvantage score (*n* = 429)

Deprivation index	Crude RR (CI 95%)	Partially^a^ Adjusted RR

Area Deprivation	1.05 (0.99, 1.11)	1.06 (1.01, 1.12)*
ROI: People	1.11 (1.00, 1.25)	1.14 (1.01, 1.30)*
Labor Market Engagement	1.07 (1.01, 1.13)*	1.08 (1.02, 1.15)*
Poverty	1.00 (0.94, 1.07)	1.01 (0.95, 1.08)
ROI: Place	0.99 (0.87, 1.13)	0.96 (0.84, 1.09)
Jobs Proximity	0.93 (0.87, 1.01)	0.93 (0.87, 1.00)*
Low transportation cost	1.00 (0.93, 1.07)	1.00 (0.94, 1.06)
School proficiency	1.07 (1.01, 1.13)*	1.07 (1.01, 1.13)*
Walkability	0.99 (0.91, 1.07)	0.98 (0.90, 1.06)
CA Healthy Places	1.06 (1.00, 1.12)	1.08 (1.01, 1.15)*
Social vulnerability	1.07 (0.88, 1.30)	1.06 (0.88, 1.28)

a Adjusted by sex, age at diagnosis, race/ethnicity, self-reported educational attainment, cancer stage and histology and smoking history

* Significant (*p*-value < 0.05)

## Data Availability

The datasets generated during and/or analyzed during the current study are available from the corresponding author on reasonable request.
