# Supplementary material for: Neighborhood disadvantage is associated with KRAS-mutated non-small cell lung cancer risk
Source: J Cancer Res Clin Oncol. 2022 Nov 16;149(8):5231–40. doi: 10.1007/s00432-022-04455-7 (PMC10322188; doi:10.1007/s00432-022-04455-7)
Supplement: Supplementary file 1 — Supplementary file1 (DOCX 18 KB) [file 432_2022_4455_MOESM1_ESM.docx]

**Supplement Table 1.** Eleven index breakdown of categories and factors included in each index.

| *Indicator* | **ADI** | **ROI: People** | **Labor** | **Poverty** | **ROI: Place** | **Jobs.**  **Prox.** | **LTCI** | **Sch. Prof.** | **Walkability** | **HPI** | **SVI** |
| --- | --- | --- | --- | --- | --- | --- | --- | --- | --- | --- | --- |
| **Economic** |  |  |  |  |  |  |  |  |  |  |  |
| Poverty/  Income | X | X |  | X |  |  |  |  |  | X | X |
| Employment/  Occupation | X | X | X |  |  | X |  |  |  | X | X |
| Job Opportunity |  |  |  |  | X | X |  |  |  |  |  |
| **Education** |  |  |  |  |  |  |  |  |  |  |  |
| Education Attainment/  Engagement | X | X | X |  | X |  |  |  |  | X | X |
| School Performance/  Resources |  |  |  |  | X |  |  | X |  |  |  |
| **Health and Disability** |  |  |  |  |  |  |  |  |  |  |  |
| Insurance and Access |  |  |  |  | X |  |  |  |  | X |  |
| Child, Teen, Prenatal, Elder Health |  | X |  |  | X |  |  |  |  |  |  |
| Disability |  |  |  |  |  |  |  |  |  |  | X |
| **Housing** |  |  |  |  |  |  |  |  |  |  |  |
| Ownership and Cost/Value | X | X |  |  | X |  | X |  |  | X |  |
| Housing Type/Infrastructure | X |  |  |  |  |  |  |  |  |  | X |
| Crowding | X |  |  |  | X |  |  |  |  | X | X |
| **Infrastructure** |  |  |  |  |  |  |  |  |  |  |  |
| Transportation Access/Cost | X | X |  |  |  |  | X |  |  | X | X |
| Commuting Behavior |  | X |  |  |  |  |  |  | X | X |  |
| Phone/Internet Access | X | X |  |  |  |  |  |  |  |  |  |
| **Environment** |  |  |  |  |  |  |  |  |  |  |  |
| Pollution |  |  |  |  | X |  |  |  |  | X |  |
| Healthy/  Unhealthy Amenities |  |  |  |  | X |  |  |  |  | X |  |
| Urban Composition |  |  |  |  |  |  |  |  | X | X |  |
| **Demographic/Household Composition** | X |  |  |  |  |  |  |  |  | X | X |
| **Social Capital** |  |  |  |  |  |  |  |  |  |  |  |
| Minority/  Linguistic Isolation |  | X |  |  |  |  |  |  |  |  | X |
| Civic Engagement |  | X |  |  |  |  |  |  |  | X |  |
| Citizenship |  |  |  |  | X |  |  |  |  |  |  |
| Mobility |  |  |  |  | X |  |  |  |  |  |  |
