# Supplementary material for: Neighborhood disadvantage is associated with KRAS-mutated non-small cell lung cancer risk
Source: J Cancer Res Clin Oncol. Author manuscript; Available in PMC 2023 Sep 28. (PMC10322188; doi:10.1007/s00432-022-04455-7)
Supplement: Supplementary file 1 [file NIHMS1904745-supplement-Supplementary_Table_1.docx]

**Supplement Table 1.** Eleven index breakdown of categories and factors included in each index.  

| *Indicator* | **ADI** | **ROI: People** | **Labor** | **Poverty** | **ROI: Place** | **Jobs.** **Prox.** | **LTCI** | **Sch. Prof.** | **Walkability** | **HPI** | **SVI** |
| --- | --- | --- | --- | --- | --- | --- | --- | --- | --- | --- | --- |
| **Economic** |  |  |  |  |  |  |  |  |  |  |  |
| Poverty/ Income | X | X |  | X |  |  |  |  |  | X | X |
| Employment/ Occupation | X | X | X |  |  | X |  |  |  | X | X |
| Job Opportunity |  |  |  |  | X | X |  |  |  |  |  |
| **Education** |  |  |  |  |  |  |  |  |  |  |  |
| Education Attainment/ Engagement | X | X | X |  | X |  |  |  |  | X | X |
| School Performance/ Resources |  |  |  |  | X |  |  | X |  |  |  |
| **Health and Disability** |  |  |  |  |  |  |  |  |  |  |  |
| Insurance and Access |  |  |  |  | X |  |  |  |  | X |  |
| Child, Teen, Prenatal, Elder Health |  | X |  |  | X |  |  |  |  |  |  |
| Disability |  |  |  |  |  |  |  |  |  |  | X |
| **Housing** |  |  |  |  |  |  |  |  |  |  |  |
| Ownership and Cost/Value | X | X |  |  | X |  | X |  |  | X |  |
| Housing Type/Infrastructure | X |  |  |  |  |  |  |  |  |  | X |
| Crowding | X |  |  |  | X |  |  |  |  | X | X |
| **Infrastructure** |  |  |  |  |  |  |  |  |  |  |  |
| Transportation Access/Cost | X | X |  |  |  |  | X |  |  | X | X |
| Commuting Behavior |  | X |  |  |  |  |  |  | X | X |  |
| Phone/Internet Access | X | X |  |  |  |  |  |  |  |  |  |
| **Environment** |  |  |  |  |  |  |  |  |  |  |  |
| Pollution |  |  |  |  | X |  |  |  |  | X |  |
| Healthy/ Unhealthy Amenities |  |  |  |  | X |  |  |  |  | X |  |
| Urban Composition |  |  |  |  |  |  |  |  | X | X |  |
| **Demographic/Household Composition** | X |  |  |  |  |  |  |  |  | X | X |
| **Social Capital** |  |  |  |  |  |  |  |  |  |  |  |
| Minority/ Linguistic Isolation |  | X |  |  |  |  |  |  |  |  | X |
| Civic Engagement |  | X |  |  |  |  |  |  |  | X |  |
| Citizenship |  |  |  |  | X |  |  |  |  |  |  |
| Mobility |  |  |  |  | X |  |  |  |  |  |  |
